# Supplementary material for: The Structure of the Drp1 Lattice on Membrane
Source: J Mol Biol. Author manuscript; Available in PMC 2026 Jan 11. (PMC12790805; doi:10.1016/j.jmb.2025.169125)
Supplement: Supplementary Data [file NIHMS2133234-supplement-Supplementary_Data.docx]

Supplementary Information


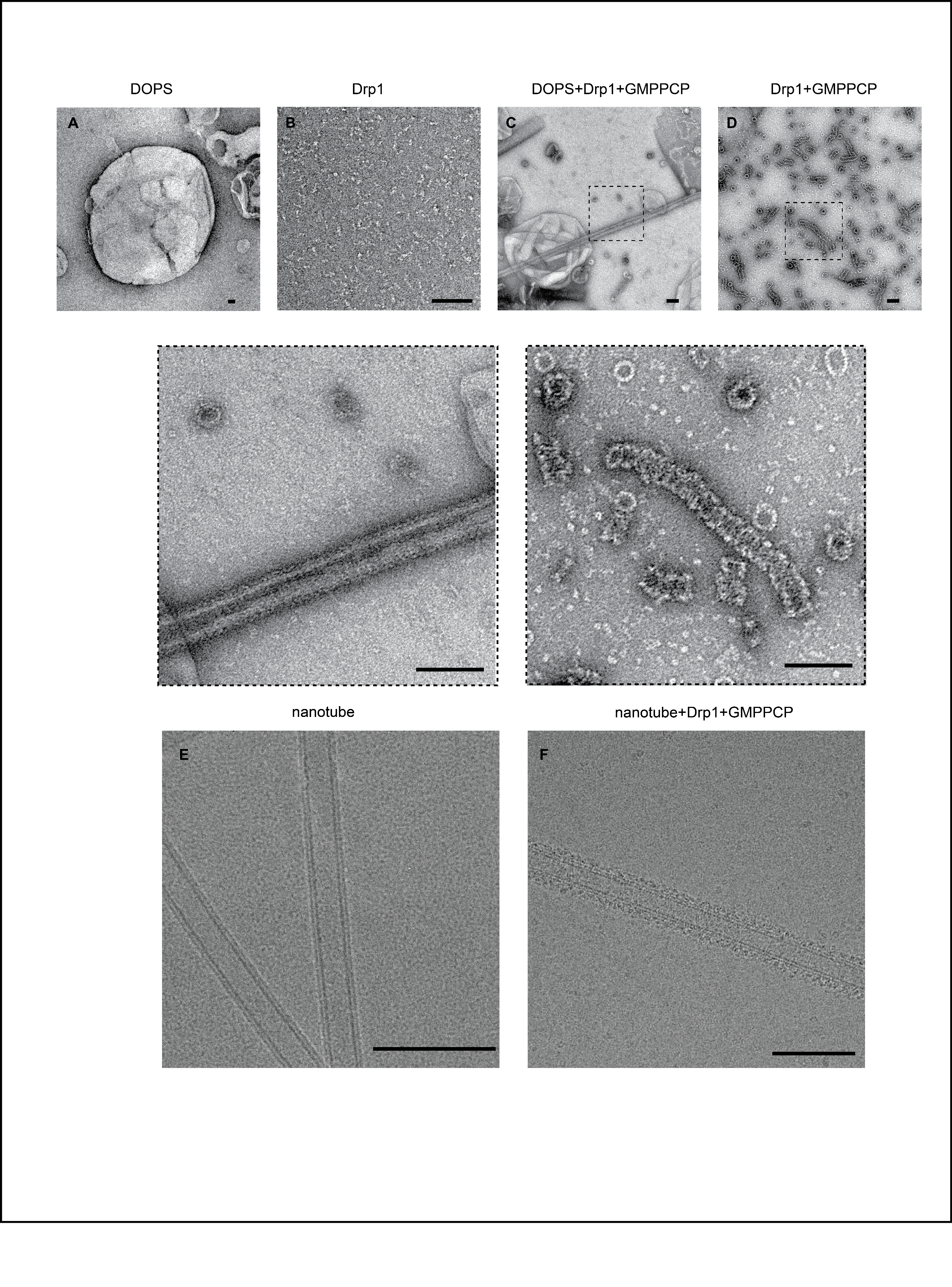


**Figure S1.** **Drp1 assembly on membrane.**  Negative staining image of DOPS liposome **(A)**, Drp1 alone **(B)** DOPS incubated with Drp1 in the presence of 1mM GMPPCP **(C)** Drp1 incubated with 1mM GMPCP **(D).** (**E)** Cryo-EM image of naked GalCer tube. (40% GalCer, 35% PE, 25% PA)**. (F)** Cryo-EM image of Drp1 decorated GalCer tube. Scale bar, 100 nm.


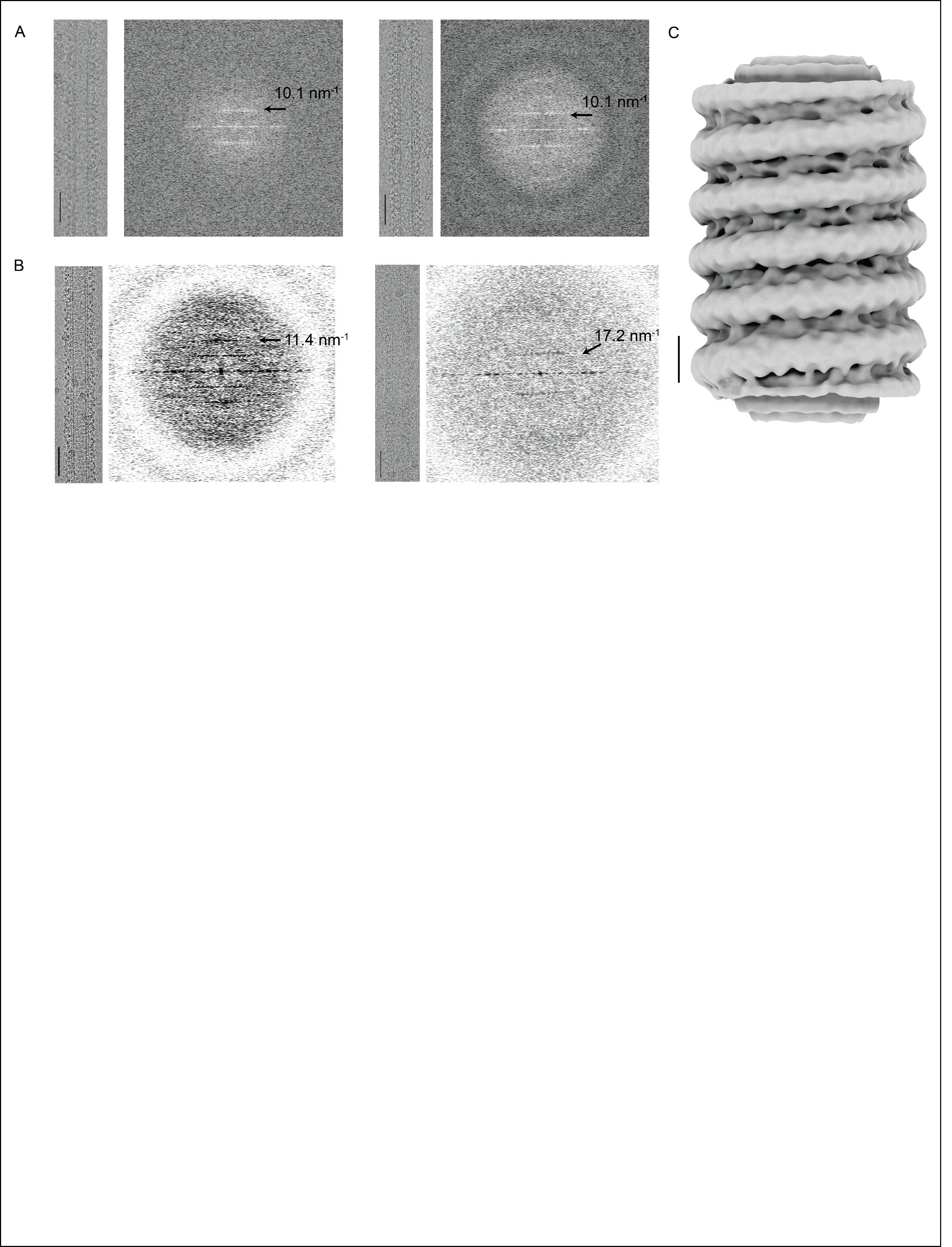


**Figure S2.** **Drp1 helical reconstruction.**  **(A)** Two representative segments of Drp1 incubated with GalCer in dataset 1. **(B)** Two representative segments of Drp1 incubated with GalCer in dataset 2. Left, segment image. Right, zoomed Fourier transform of the segment. **(C)** Helical reconstruction using helical symmetry parameters estimated in Fig. 1B. Scale bar, 50nm for A, B. Scale bar, 20nm for C.


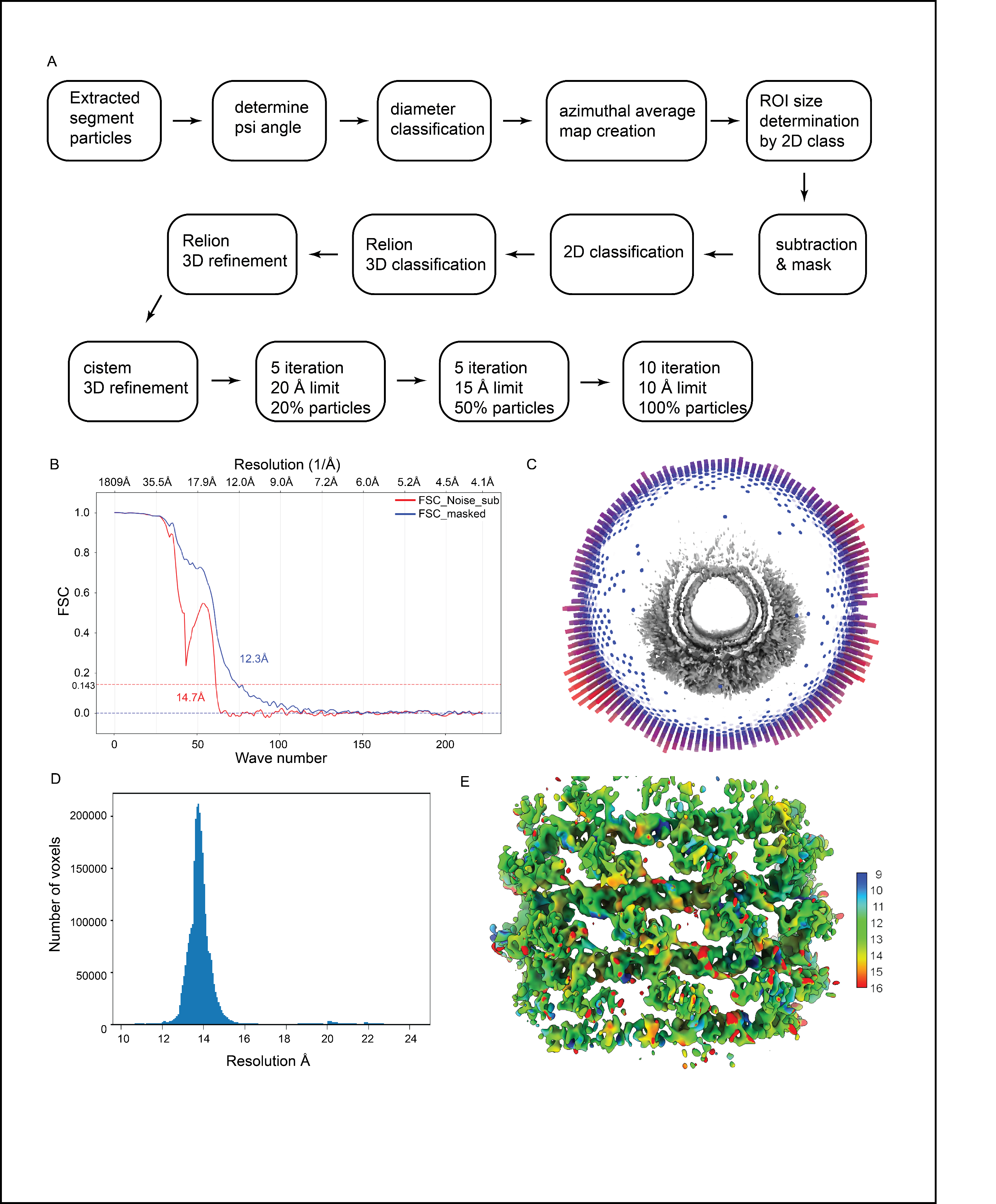


**Figure S3. Data processing of stacked state Drp1 lattice**. **(A)** Workflow of data processing. **(B)** FSC curve of stacked state Drp1 lattice reconstruction. Red, phase randomized FSC. Blue, FSC with mask of protein lattice shape. **(C)** Angle distribution of stacked state Drp1 lattice reconstruction. **(D)** Local resolution counts of stacked state Drp1 map. **(E)** Stacked state map colored by local resolution index.


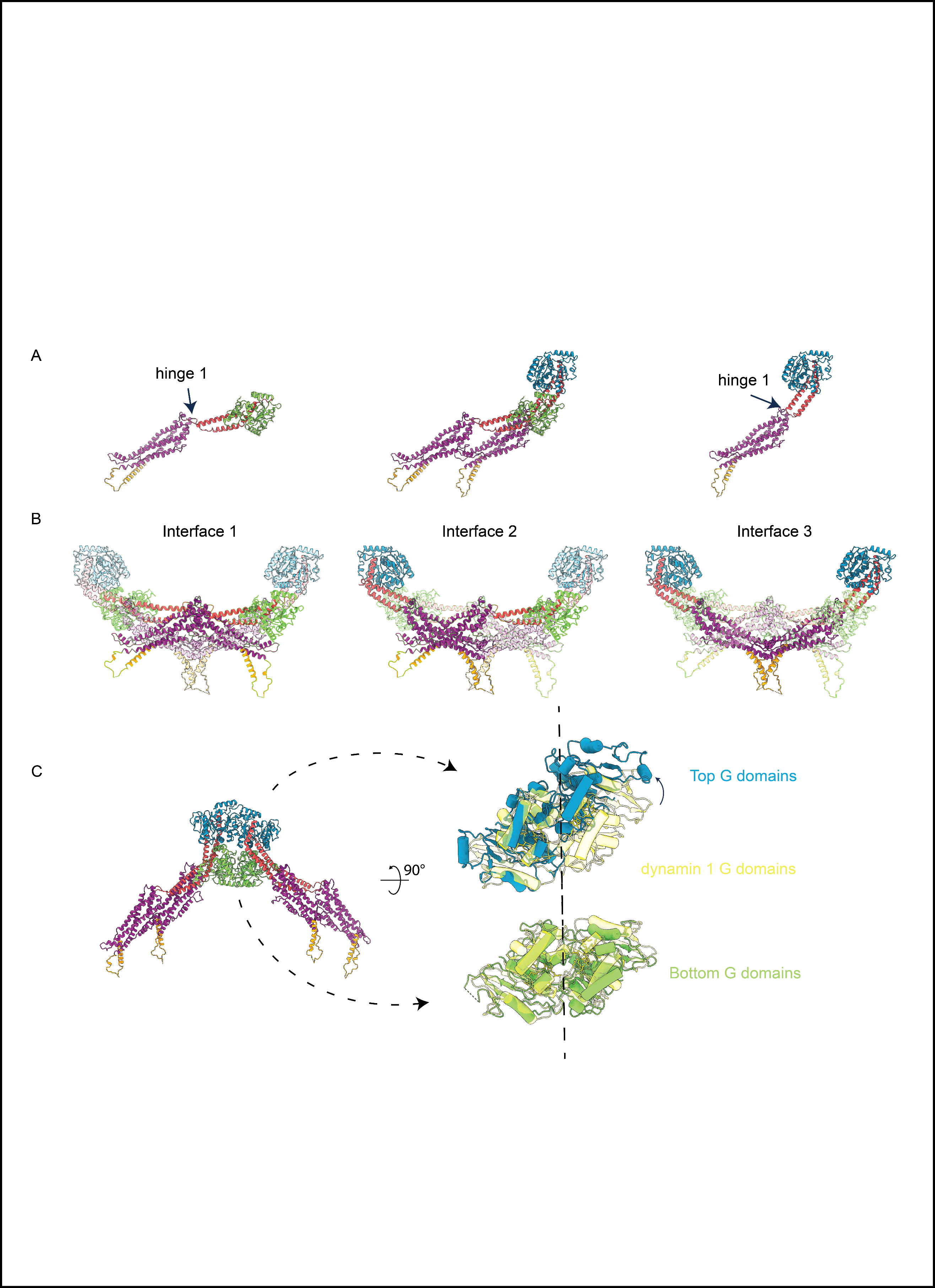


**Figure S4.** **Model details of stacked state Drp1 lattice.**  **(A)** Poses between stalk domain and BSE. Green G domain represent extended conformation. Blue G domain represent contracted conformation. **(B)** Interfaces of stalk domains. Left, interface 1. Middle, interface 2. Right, interface 3. **(C)** GG interface of stacked state Drp1 lattice. Right images are taken as looking from outer radial position to the center of tubules. Transparent yellow represent GG domains in dynamin 1 (PDB ID: 6DLU). Left G domains are fitted together.


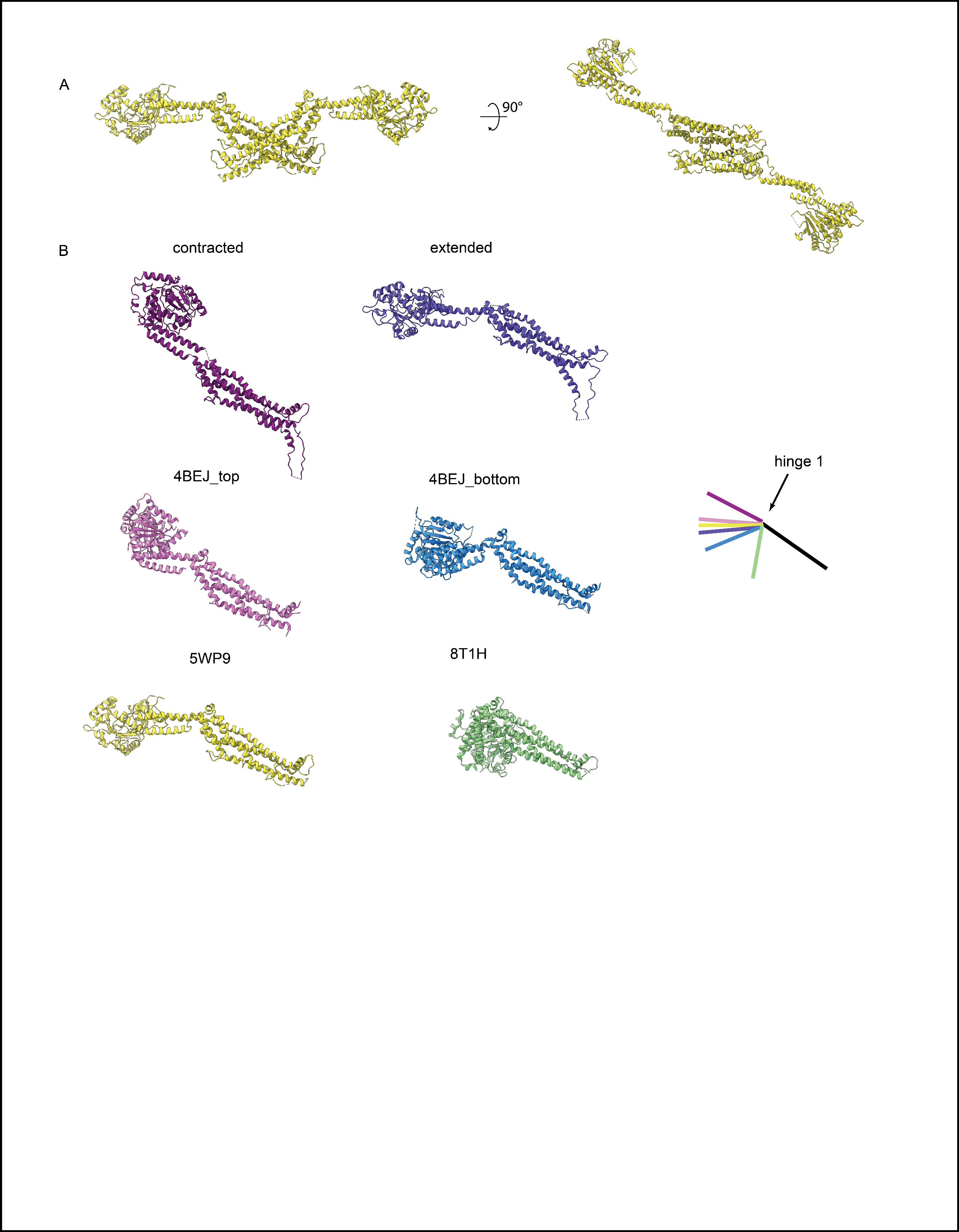


**Figure S5.** **Hinge 1 conformation comparison.**  **(A)** 5WP9 dimer, showing orientations taken for comparison. **(B)** Hinge 1 conformation comparison. All models are superimposed on stalk region.

**
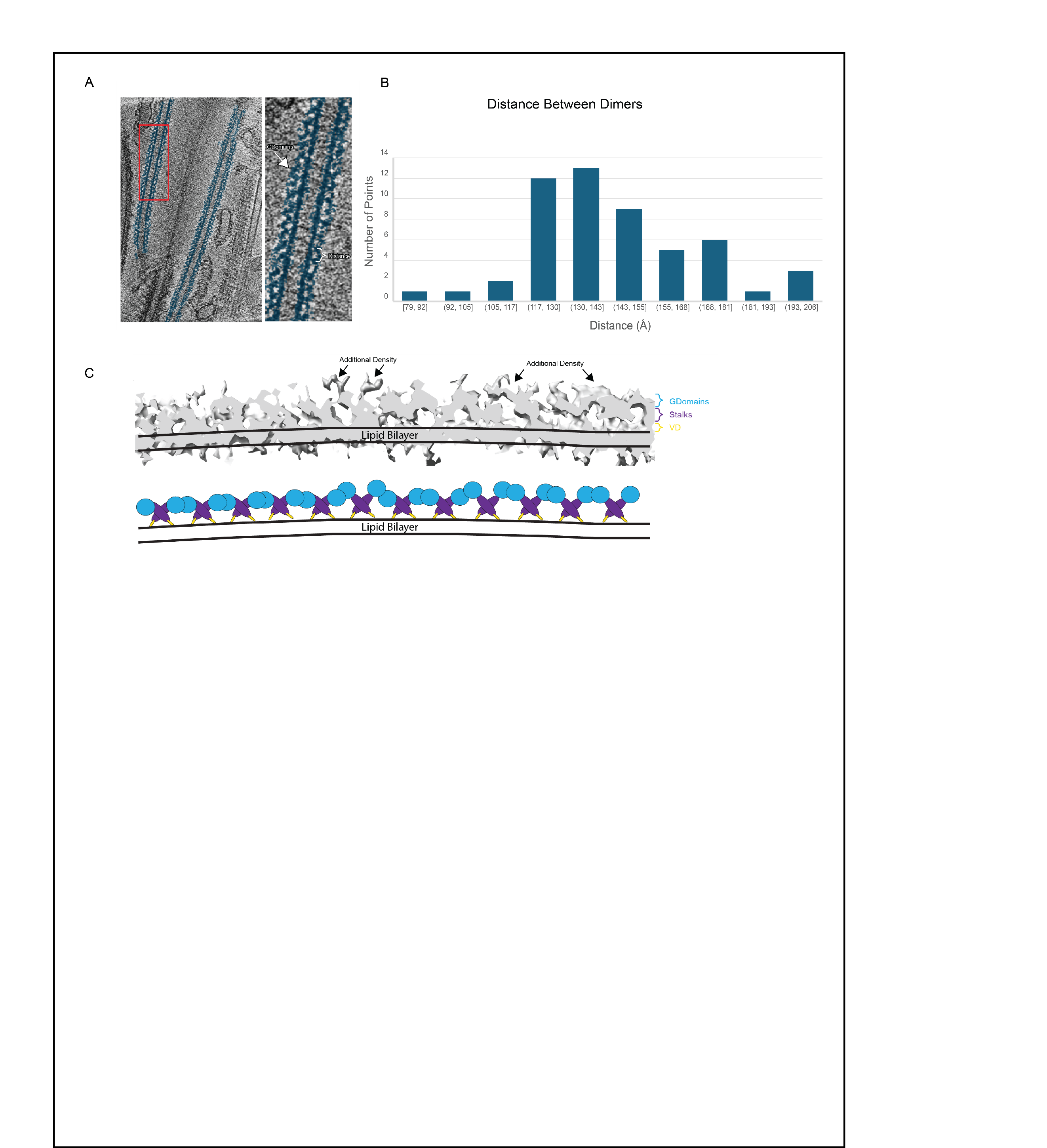
**

**Figure S6. Cryo-ET on Drp1 decorated GalCer tubes.** **(A)** Tomography of Drp1 decorated GalCer. Protein features colored blue. **(B)** Distance quantification of tomography reconstruction. Distance is defined as the spacing between adjacent stalks. **(C)** Tomography map and schematic models.

Supplementary Table 1. Cryo-EM data collection, refinement, and validation statistics

|  | Drp1 Asymmetric Tetramer  (EMDB-43045)  (PDB 8V8T) |
| --- | --- |
| **Data collection and processing** |  |
| Voltage (kV) | 300 |
| Electron exposure (e–/Å^2^) | 58.49 |
| Defocus range (μm) | -0.8 to -2.0 |
| Pixel size (Å) | 2.02 |
| Symmetry imposed | C1 |
| Initial particle images (no.) | 51,102 |
| Final particle images (no.) | 13,215 |
| Map resolution (Å)  FSC threshold | 11.37  0.143 |
|  |  |
| **Refinement** |  |
| Initial model used (PDB code) | 4BEJ, 5WP9 |
| Model composition  Non-hydrogen atoms  Protein residues  Ligands | 22,382  2918  0 |
| R.m.s. deviations  Bond lengths (Å)  Bond angles (°) | 0.003  0.706 |
| Validation  MolProbity score  Clashscore  Poor rotamers (%) | 2.81  12.51 |
| Ramachandran plot  Favored (%)  Allowed (%)  Disallowed (%) | 89.09  9.64  1.27 |
